# Possibly pathogenic bacteria in aerosols and foams as a result of aeration remediation in a polluted urban waterway

**DOI:** 10.1007/s12223-023-01096-2

**Published:** 2023-09-30

**Authors:** Joby Jacob, Ingrid Veras, Olga Calderόn, Holly A. Porter-Morgan, Joshua Tan, Harry E. Aguilar, Willis T. Elkins, Veronica P. Martinez Castro, Vania Fulton, Wesam K. Yousri

**Affiliations:** 1 Natural Sciences Department, LaGuardia Community College, City University of New York, Long Island City, NY USA; 2 Newtown Creek Alliance, Brooklyn, NY USA

**Keywords:** 16s rRNA, Foam, Aeration, Water remediation, Newtown Creek - Hudson River Estuary - New York – USA, Bacterial pathogens

## Abstract

Newtown Creek is a tributary of the Hudson River Estuary. It has a legacy of both industrial pollution and sewage pollution and has been designated a Superfund site. To ameliorate the chronically low levels of dissolved oxygen detected in the Creek, the New York City Department of Environmental Protection has been installing aerators. The abundance of various bacteria in the aerosols, foams, and water, at two sites in the Creek, was studied before, during, and after the aeration process. Additionally, aerosols and dispersed foams created by the aeration process were sampled and cultured to determine what unique taxa of bacteria could be grown and identified. Taxa including Actinobacteria and Firmicutes were prevalent in cultures taken from aerosols, whereas Gammaproteobacteria were prevalent in cultures taken from foam. Campylobacteria was found to have a significant presence in both samples taken after the aerators were turned off. These taxa include potentially pathogenic bacteria and are therefore of particular concern.

## Introduction

Newtown Creek is a 6.1-km-long tidal waterway that flows between the boroughs of Brooklyn and Queens in New York City. It is a Tier 2^1^ sediment contaminated site in the middle of the most densely populated city in the USA, surrounded by growing residential neighborhoods. The community districts that surround the Creek have a combined residential population of over 600,000 people (New York City Department of City Planning 2021). The Creek was added to the National Priorities List and designated a Superfund site in 2010 (City of New York Department of Environmental Protection 2011).

Newtown Creek is a tributary of the East River and is part of the larger Hudson River Estuary. There are very few natural areas along the Creek as much of the surrounding region was rapidly developed. In the nineteenth century, the Creek was transformed into an industrial waterway after being widened, dredged, and bulkheaded. By the twentieth century, the Creek was heavily polluted by several hazardous substances (Newtown Creek Alliance nd). Currently, many of the areas surrounding the Creek are subject to mixed-use residential and commercial rezoning and redevelopment (New York City Department of City Planning 2018).

The waterway is also severely impacted by twenty-two combined sewer overflow (CSO) outfall pipes that deliver over 5.46 billion liters of raw sewage, wastewater, and street runoff every year into the waters of Newtown Creek. This level of CSO input contributes high levels of nutrients into the waterway which promotes algal blooms and eutrophication and leads to fluctuations in dissolved oxygen (DO) levels (2017). The upstream areas of the Creek (where samples for this study were collected) have elevated levels of polychlorinated biphenyls, polycyclic aromatic hydrocarbons, and heavy metals (The United States Department of the Interior, New York State Department of Environmental Conservation, and National Oceanic and Atmospheric Administration in their capacity as Trustees of Natural Resources 2012). Additionally, exposure to aerosols due to aeration may be a potential health risk (Pendergraft et al. 2021).

Aeration is an effective way of increasing DO in an anoxic or oxygen-deprived water body and has been used on the Flambeau River in Wisconsin since 1943 (Alp and Melching 2011). In 2008, the New York City Department of Environmental Protection (DEP) began installing a bottom-up in-stream aeration system. These types of aerators employ pipes sited on cinderblocks that lie on the Creek’s soft sediment through which air escapes and bubbles to the surface. These aerators typically operate between May and September, to address the long-recognized problem of dangerously anoxic levels in Newtown Creek due primarily to eutrophication.

Installation of aerator pipes may exacerbate the rate of mixing and aerosol formation, as well as release and dispersal of aerosols. The dispersal rate of aerosols produced by aeration pipes can additionally be influenced by channel geometry and water velocity (King 1970). Aerosol particulate size and diversity may also be impacted by humidity, temperature, wind speed, and direction. Thus, strong winds and gusts can transport larger aerosolized particles further inland resulting in changes to microbial communities far away from the aerators (Dueker et al. 2018). Other findings demonstrate that aerosols can travel up to 720 m inland from the source suggesting potential impacts to public health from aerosols (Pendergraft et al. 2021). A 2014 study of the aerators located on the English Kills branch of Newtown Creek (which is further upstream of the sites used in this study) demonstrated that aeration increases the levels of oxygen in the Creek; however, it also increases the amount of water-associated bacteria that are aerosolized during the process, including *Vibrio* (Dueker and O’Mullan 2014).

Comparative studies of urban and non-urban water bodies and aerosolized bacteria suggest that besides atmospheric conditions, anthropogenic sources can also influence the composition of urban bacterial diversity (Dueker et al. 2018). For over a decade, studies have demonstrated that microorganisms are aerosolized adjacent to aerator pipes (Dueker et al. 2012, 2018; Dueker and O’Mullan 2014; Montero et al. 2016). These findings warrant further study as urban waterways are often contaminated with sewage, and sewage-associated bacteria can become aerosolized (Dueker et al. 2018).

The aeration system at Newtown Creek may affect not only aerosolization but also the production of foam on the surface of the water. Foam can be formed naturally in aquatic environments where water and organic compounds, such as surfactants, are disturbed by wind and currents. Furthermore, foam formation is observed when sewage is aerated (Collivignarelli et al. 2020). Although foam can be observed at many locations on Newtown Creek, a noteworthy quantity of foam can be observed both in channel, above the aerator pipes, and along the shore near the aerators.

A prior study of surface water at three sites near the Dutch Kills, Whale Creek, and Maspeth Creek tributaries, which are further downstream on Newtown Creek, revealed that over 1000 bacterial species are present in the Creek, including many known animal and human pathogens. Bacterial diversity at the three sites changed after a rain event; however, the extent may be a function of the distance from CSO outfalls (Calderon et al. 2017).

New residential development near the Creek has increased demand for recreational access to the water. People living and working near the aerators may be exposed to aerosols containing sewage-associated bacteria. Along the shore, humans and other animals may come into contact with bacteria that become trapped and concentrated in the foam. We undertook this study to determine the abundance of potentially pathogenic bacteria in aerosols and persistent foams generated by the aeration process and answer community questions about whether there is potential for exposure to these pathogens.

In this study, we performed 16S rRNA amplicon-based next-generation sequencing to characterize the microbiota of the Creek as well as aerosols and foams produced as a result of aeration remediation. We also examined our samples to (i) reveal taxonomic differences, (ii) examine the relative abundance of the taxa, (iii) characterize the differences in taxa when the aerators were on or off, and (iv) characterize the differences in taxa in the cultures grown from the foam and the aerosols produced by aeration.

## Materials and methods

### Site selection and description

**Fig. 1:**
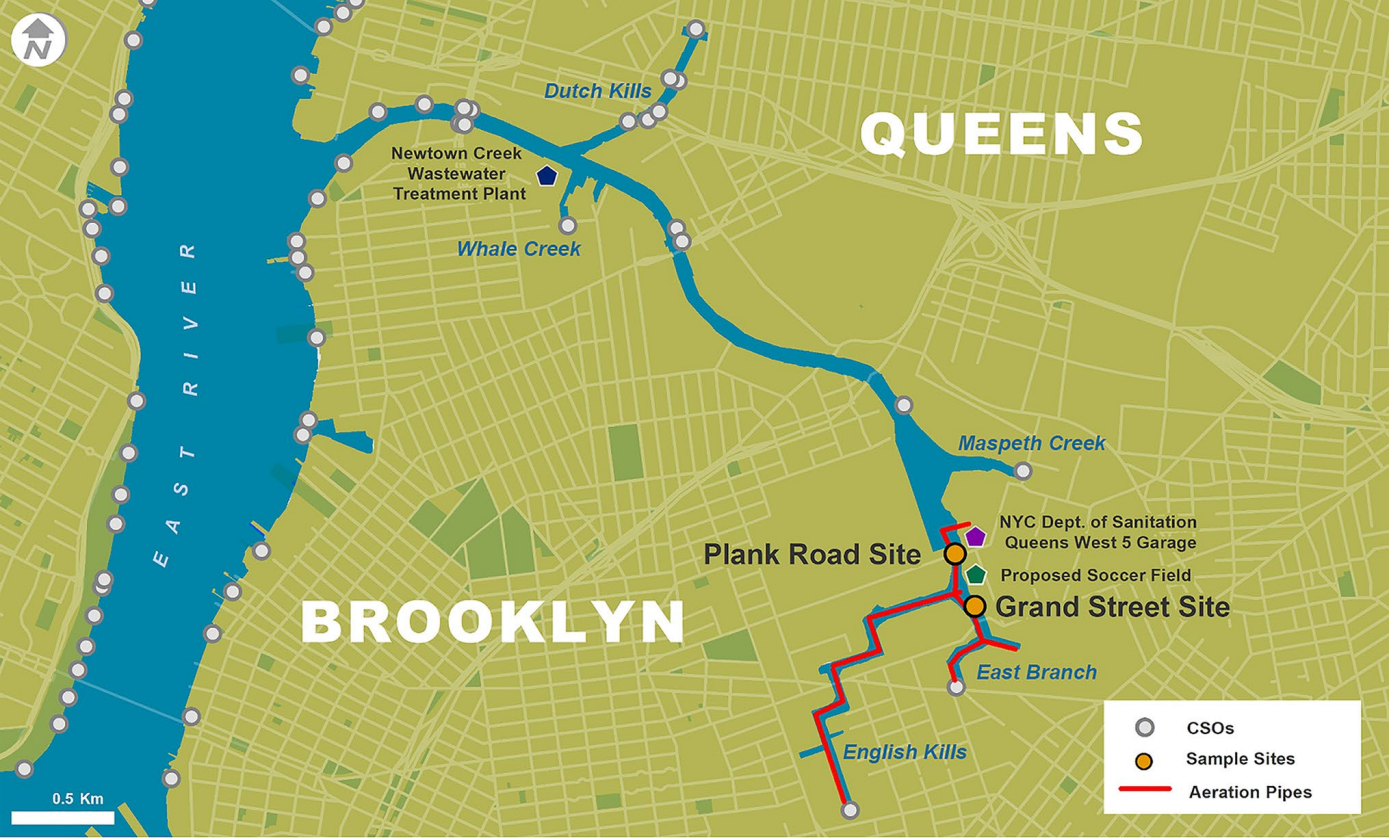
Newtown Creek, aerators, sampling sites, and CSO locations. Data sources: US Census Bureau, 2015, and New York State Department of Environmental Conservation (NYS-DEC), 2018

The aim of the study was to identify bacterial taxa with an eye towards potential pathogenicity and their presence in various conditions associated with aeration. Samples were collected at two sites, Plank Road (40.720° N, 73.924° W) and Grand Street (40.717° N, 73.923° W) (Fig. 1). The two sites differ in terms of depth, motorized boat traffic, and stormwater runoff. The Plank Road site is deeper than the Grand Street site and has a more naturalistic shoreline (no bulkhead). This site is near a commercial fill distributor, a concrete plant, several food-related businesses, a NYC Sanitation facility, and the National Grid Greenpoint Energy Center which is a former manufactured gas plant (National Grid 2012). Additionally, the Plank Road site is traversed by more passing vessels, such as tugboats, in comparison to the Grand Street site. The Grand Street collection site has a bulkhead shoreline and is near a bridge pier, a concrete plant, a bus terminal, and a truck parking lot. Both collection sites have documented coal tar contamination (U.S. Environmental Protection Agency-Region 2016) and are located above aeration pipes in the waterway. The Grand Street site is 350 m away and the Plank Street site is 701 m away from the nearest CSO outfall (NCB-083) which discharges over 1.36 billion liters per year (City of New York Department of Environmental Protection and Bureau of Engineering Design & Construction 2017). Of concern at both sites, the propeller wash from passing large vessels may cause disturbance and resuspension of surface sediments, and the wake of these vessels may enhance the formation and dispersion of the foam.

### Sample collection and genomic DNA extraction

Surface water samples were collected at various times over 2 years: before the aerators were turned on (5/31/2019), during their operation (07/15/2019 and 09/29/2019), and after they had been turned off (04/23/2022). Additionally, on 09/29/2019, aerosol and foam samples were collected at these same sites. For the samples, there was no precipitation for at least 2 days prior to each sample collection, except for the samples collected on 05/31/2019 which happened after 2 days of precipitation, which likely means there was a CSO event. Water quality was measured within two-to-3 days of each sample collection and the did not vary significantly (Billion Oyster Project 2023). On the day of aerosol collection (09/29/2019), wind direction was north–northeast and wind speed was 0–8.5 m/s with gusts of 10.3 m/s.

Because the aerators are forcing air into the bottom of the Creek, bubbles of air rise to the surface and produce aerosols and what we are terming “aerator foams” (which are non-persistent foams that are found at the aeration site itself). Samples were collected at the aerators in 50-mL conical tubes from a boat located above the aeration pipes at the Plank Road and Grand Street sites. Tubes were submerged sideways 5 cm below the water’s surface to collect surface water samples and tubes were skimmed along the surface to aerator foams. Due to personnel and resource constraints, the samples could not be processed immediately after collection and therefore had to be stored at –20 °C until genomic DNA isolation. Additional sample replication was not also not possible due to these externalities.

**Fig. 2:**
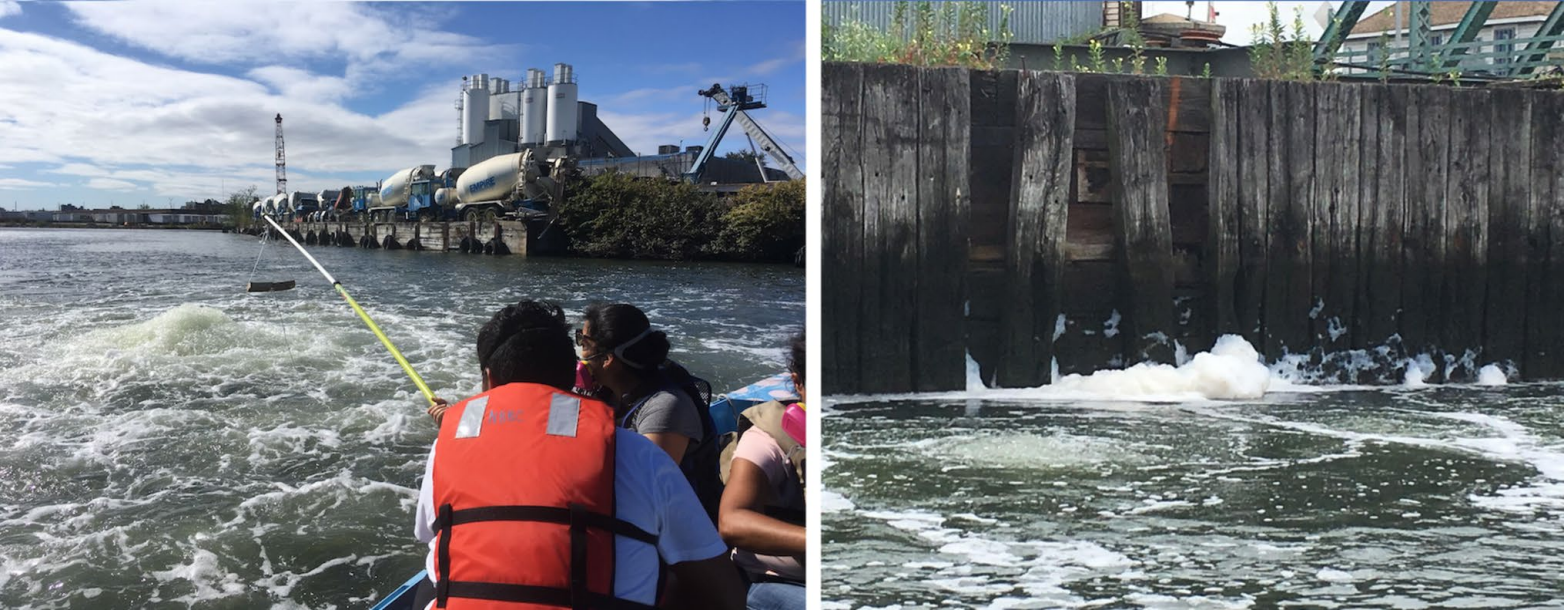
**a** Castro and Aguilar demonstrating apparatus used to capture the aerosols. **b** An aerator in the foreground and the dispersed foam along a bulkheaded section of the Creek in the background

Bacterial aerosol samples were collected utilizing petri dishes with Marine Agar (BD Difco Marine Agar 2216) and Tryptic Soy Agar (BD Difco Tryptic Soy Agar (Soybean-Casein Digest Agar)) plates. To minimize environmental contamination, sterile media were attached with a hook-and-loop tape (Velcro) to an upside-down cardboard box, which was hung from a painter’s pole, and the plate lids were kept closed and opened before collecting aerosols. The sides of the box extended approximately 5 cm and helped to prevent wind-borne bacteria from contaminating the plates. A rope attached to the box helped ensure that aerosols could be collected at the same height in different locations (see Fig. 2a). The plates were suspended 60 cm above the water surface at sample sites for 5 min (Fig. 2a). After the sample was collected, the petri dishes were covered immediately, detached, and stored in sterile bags. The plates were taken to the laboratory and incubated for 3 days in conditions as described below.

One of the authors (Elkins) has been consistently monitoring environmental conditions on the Creek and has noted a persistent and large quantity of what we term “dispersed foam” which is only present when the aerators are functioning. This foam is different from the aerator foam found at the surface of the water above aeration pipes. Instead, this dispersed foam is dispersed by water and wind movement towards the shoreline (see Fig. 2b) and remains persistent. Dispersed foam samples were collected at the shore adjacent to the Plank Road site and along the bridge pier near the Grand Street site at the coordinates indicated above. Samples of both foam types were collected utilizing 50-mL sterile conical centrifuge tubes. The dispersed foam samples were immediately taken to the laboratory and spread on plates utilizing a sterile swab. These plates along with the plates used to collect aerosols were then incubated for 3 days in conditions as described below.

A list of the samples collected and tested with cultivated plates aggregated by site and sample type are presented in Table 1. The cultivated plates were grown in all possible combinations of the following variables—temperature (25 °C or 37 °C), media (Tryptic Soy Agar or Marine Agar), and oxygen conditions (aerobic or anaerobic). To prepare for genomic DNA extraction, sterile PBS was added, and the colonies were scraped with a sterile cell scraper, collected into a sterile 15-mL conical tube, and vortexed to yield 3 mL of homogeneous mixture. Four hundred microliters of the lysate was used downstream in genomic DNA extraction. Table 1 also shows the conditions under which in situ samples were collected.

**Table 1:** Samples collected and processed for genomic DNA

#	Site	Sample type	Date collected	Aerator status	In situ (I) or cultivated (C)
GW-A	Grand St	Surface water	05/31/2019	Off	I
PW-A	Plank Rd	Surface water	05/31/2019	Off	I
GW + A	Grand St	Surface water	07/15/2019	On	I
PW + A	Plank Rd	Surface water	07/15/2019	On	I
GW + B	Grand St	Surface water	09/29/2019	On	I
PW + B	Plank Rd	Surface water	09/29/2019	On	I
GW-B	Grand St	Surface water	04/23/2022	Off	I
PW-B	Plank Rd	Surface water	04/23/2022	Off	I
PF + B	Grand St	Aerator foam	09/29/2019	On	I
GF + B	Plank Rd	Aerator foam	09/29/2019	On	I
GA	Grand St	Aerosols	09/29/2019	On	C
PA	Plank Rd	Aerosols	09/29/2019	On	C
GD	Grand St	Dispersed foams	09/29/2019	On	C
PD	Plank Rd	Dispersed foams	09/29/2019	On	C

Genomic DNA (gDNA) was extracted with Zymo Research Quick-gDNA MiniPrep Kit (Irvine, CA) as per the manufacturer’s protocol from the tubes containing aerator foams/surface water samples and tubes containing cell scrapings from aerosol/dispersed foam plates.

The study design was meant to approximate the conditions where pathogens would be incubated after exposure to aerated and dispersed material. Thus, the priority was plating aerosols and dispersed foams and culturing them in conditions that could indicate which bacteria are able to grow. Owing to resource constraints, similar procedures were not possible with surface water samples and aerator foams which were, nevertheless, sampled and processed for genomic sequencing.

### 16S rRNA sequencing and bioinformatic analysis

Genomic DNA was sent to Azenta (South Plainfield, NJ) for Illumina MiSeq PE 2 × 250 bp Next-Generation DNA sequencing of the 16s rRNA targeting the V3 and V4 hypervariable regions (Qiao et al. 2018). Amplification of V3 and V4 regions was done using forward primers containing sequence “CCTACGGRRBGCASCAGKVRVGAAT” and reverse primers with sequence “GGACTACNVGGGTWTCTAATCC.” The effective sequences were used in the final analysis. Sequences were clustered with QIIME 2 (Bolyen et al. 2018) into amplicon sequence variants (ASVs) using VSEARCH (2.0) with de novo clustering (Rognes et al. 2016) performed at 97% identity, and sequences with only one match were rejected. Chimeras were removed by means of the UCHIME detection program (Edgar et al. 2011). The resulting ASVs were classified using the standard Scikit-learn classifying algorithm (Pedregosa et al. 2012) trained with the SILVA rRNA database (Quast et al. 2013) chosen to avoid specific problems documented with the Greengenes database (Lydon and Lipp 2018). The resulting datafiles and feature tables with classifications are available through the Zenodo repository: https://doi.org/10.5281/zenodo.8277796 (Jacob et al. 2023). Linear discriminant analysis (LDA) of effect size (LEfSe) was used for identifying ASVs with statistical difference between the different groups using relative abundance (Segata et al. 2011).

## Results

Table 2 indicates the Shannon diversity index, (*H*′) which conveys the quality of the sequencing and can be used to compare this study and studies of similar systems. *H*′ was calculated using the full list of ASVs in each sample. Unsurprisingly, from Table 2, the cultured samples have a lower diversity in general than the in situ samples. But a higher sample taxonomic diversity does not indicate the potential presence of pathogens. We elaborate more in the “Discussion” section.

**Table 2:** Samples collected and processed for genomic DNA

#	GW-A	PW-A	GW + A	PW + A	GW + B	PW + B	GW-B	PW-B	PF + B	GF + B	GA	PA	GD	PD
*H*′	6.82	7.12	6.87	7.68	5.91	4.05	6.06	6.05	5.88	7.38	4.09	4.88	5.05	4.15

Table 3 presents selected taxa mentioned in this paper, documenting their absolute abundance in the samples described in Table 1. In cases where more specific taxonomies are resolved, those abundances are also included in the higher-level taxonomies. Relative abundances for any given sample can be calculated by dividing by the value of the last row of any column which is the total count of all sequenced ASVs in the sample. Note that the final four columns are the aggregate data of eight different samples taken from their respective sites and cultured under the conditions described in the “Materials and methods” section.

**Table 3:**
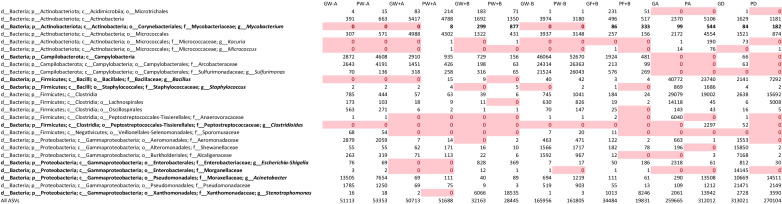
Absolute abundance of bacteria in samples. Relative abundance of selected ASVs from bacteria collected in situ (GW-A, PW-A, GW + A, PW + A, GW + B, PW + B, GW-B, PW-B, GF + B, PF + B) and cultured from aerosols (GA, PA) and dispersed foams (GD, PD) collected and grown in the laboratory. Highlighted cells have a value of zero and the last row is the cumulative number of samples that were successfully clustered

### Relative abundance of bacteria in surface water

**Fig. 3:**
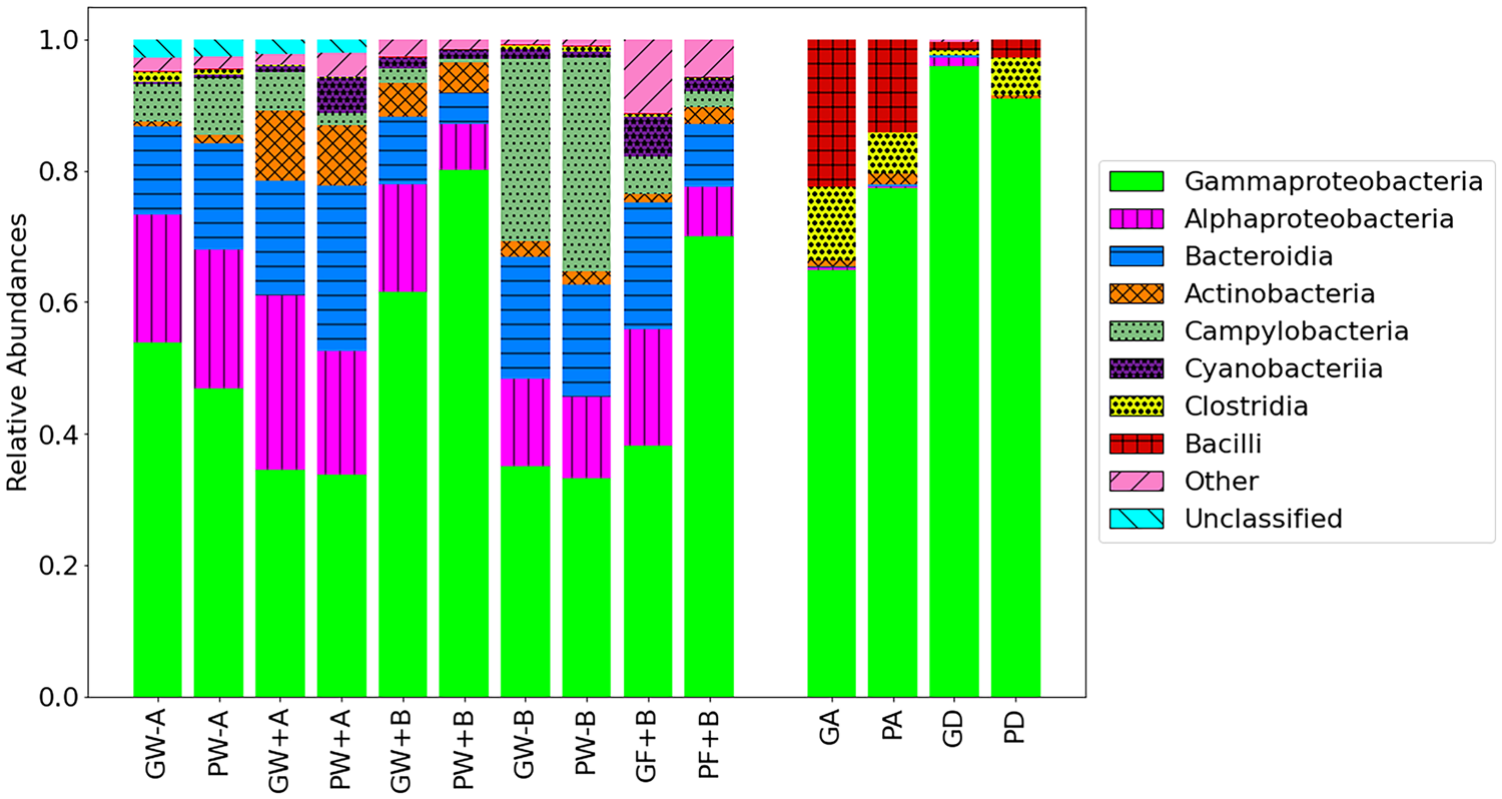
Characterization of surface water microbiota. Microbial community relative abundance bar plot of the eight most abundant classes in surface water and cultured samples collected from aerator sites. Labels correspond to those indicated in Table 1

In the surface water samples collected in situ from the two sites (Fig. 3), the relative abundance of the phylum Actinobacteria is notably higher in surface water when the aerators are on (GW + A, PW + A, GW + B, PW + B) versus when they are off (GW-A, PW-A, GW-B, PW-B). The relative abundance of Actinobacteria is also low in aerator foams collected in situ (GF + B, PF + B). There was a low relative abundance of both classes Bacilli and Clostridia (phylum Firmicutes) in all surface water and aerator foams, but the relative abundance is noteworthy in the cultured samples (GA, PA, GD, PD). There was a high relative abundance of the class Gammaproteobacteria (phylum Proteobacteria) in all samples. The relative abundance of the class Campylobacteria (phylum Campylobacterota) is higher in samples collected in 2022 (GW-B, PW-B) when the aerators were off compared to the other samples.

LEfSe was conducted to examine the differences between the surface water collected before the aerators were turned on (Off19), during aeration (On19), and post aeration (Off22). Statistically significant results include the following:

- Higher relative abundance of the phylum Firmicutes in samples collected prior to aeration in 2019 (GW-A, PW-A). Note that a CSO event may explain this result.
- Higher relative abundance of the class Actinobacteria (phylum Actinobacteriota) when the aerators were on (GW + A, PW + A, GW + B, PW + B).
- Higher relative abundance of class Campylobacteria (phylum Campylobacterota)in samples collected after the aerators had not been turned on for 2 years (GW-B, PW-B).

### Abundance of bacteria in cultured samples

LEfSe was also used to determine which taxa most significantly contributed to the differences between the aerator and dispersed foam plates. The 20 taxa which had an LDA score greater than 2 are shown in Fig. 5. The class Gammaproteobacteria and the constituent orders Pseudomonadales and Aeromonadales and the families Morganellaceae, Alcaligenaceae, and Shewanellaceae were at statistically significant higher relative abundance in the dispersed foams whereas the taxa with the most statistically significant high relative abundance for the aerosols included the phyla Actinobacteriota and Firmicutes and their constituent classes Micrococcales and Bacillaceae respectively.

### Potential pathogens

Taxa found in abundance that include potential pathogens are the genus *Mycobacterium*, the class Campylobacteria, the genus *Bacillus*, the genus *Clostridioides*, the genus *Escherichia*, the family Morganellaceae, the genus *Acinetobacter*, and the genus *Stenotrophomonas*. These taxa are bolded in Table 3. While in some cases, there was not enough evidence to conclude that the presence of these taxa was significantly affected by changing conditions, in cases where there was strong evidence, analysis of the implications is provided in the “Discussion” section.

## Discussion

The two questions we aimed to answer with this study were (a) which bacterial taxa were more abundant when aerators were turned on versus when they were off? and (b) which bacterial taxa were more abundant in the dispersed foams versus aerosols? with an eye towards potential pathogenicity. Cultured plates were used to identify taxa which could colonize new environments after exposure to aerosols or dispersed foams including the possibility of pathogens infecting human interlocutors. The Shannon diversity index (*H*′) (Table 2) does not help answer our study questions. There are no systematic effects seen in *H*′ when considering questions (a) and (b). Rather, to answer these questions in this discussion, we identify those taxa which show increased abundance under various conditions and comment on the possibilities that such bacteria may be pathogenic.

### Taxa in different aeration conditions

**Fig. 4:**
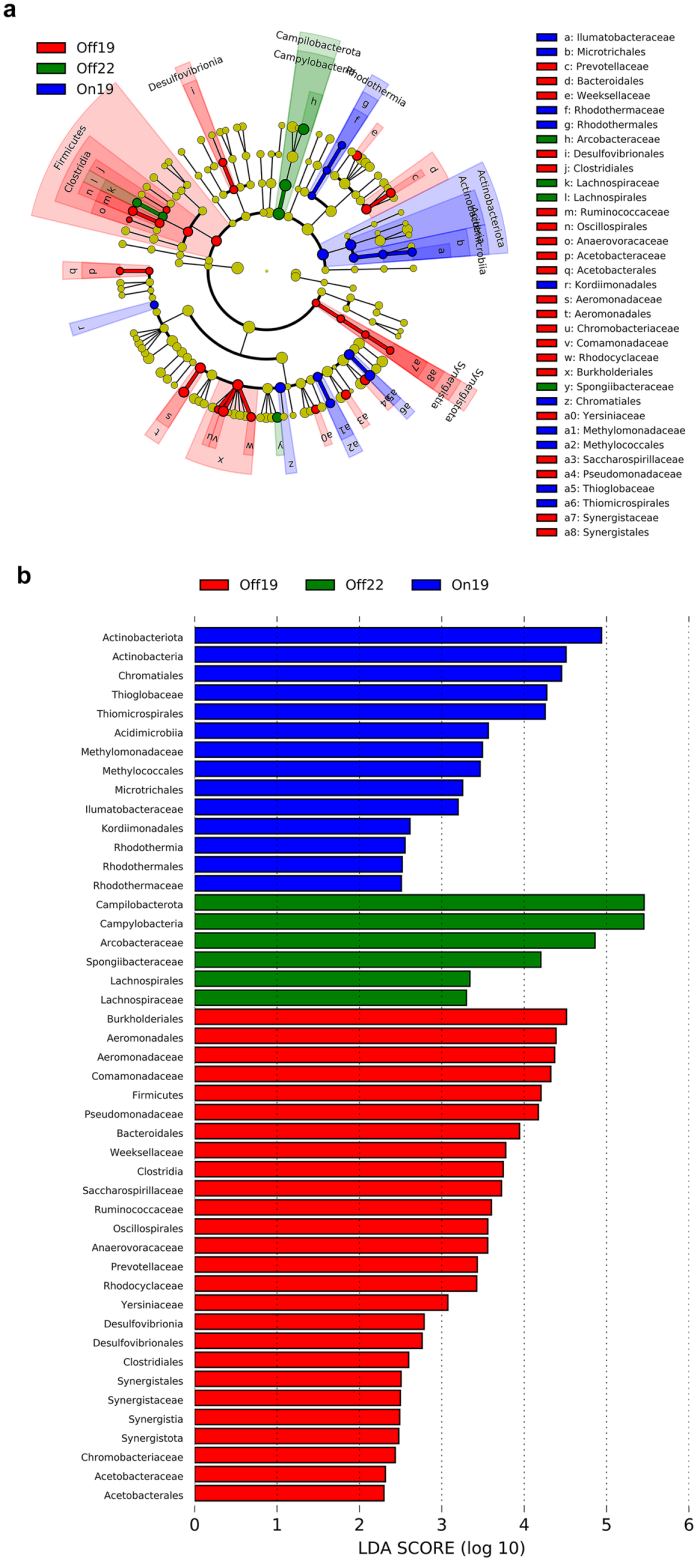
Linear discriminant analysis of effect size of the surface waters collected at the aerator sites. **a** Taxonomic representation of differential abundances of bacteria in water collected prior to the aerators being turned on in 2019 (Off19), when aerators were on (On19), and when they had been turned off (Off 19) (LDA score > 2). **b** Histogram of LDA scores ranked by effect size

Collectively, Firmicutes and Campylobacteria were relatively more abundant when the aerators were off and Actinobacteria was more abundant when the aerators were on (see Fig. 4). Detailed analysis is included below.

#### Actinobacteria

As can be seen in Fig. 3, one of the most significant effects observed is the increase in the relative abundance of the phylum Actinobacteria during aeration (GW + A, PW + A, GW + B, PW + B) compared to times when the aerators were off (GW-A, PW-A, GW-B, PW-B). LDA showed that this difference was statistically significant (Fig. 4). The order Microtrichales is indicated as one of the Actinobacteria which is significantly more abundant in surface water samples collected when the aerators are on versus when the aerators are off (Fig. 4). Members of this taxa have been previously identified as benthic metabolically flexible habitat generalists capable of adapting to multiple environments (Chen et al. 2021). This suggests the presence of a benthic bacterium that is being brought up into the surface water column by the disruption of sediment by the aeration process but which does not become part of the microbial community. While the primary order and family present are implicated in recycling organic compounds (Miksch et al. 2021), some other Actinobacteria are pathogens (Lewin et al. 2016).

#### Firmicutes

Within the phylum Firmicutes, LDA showed that the abundances of the family Sporomusaceae and the orders Lachnospirales, Oscillospirales, and Anaerovoracaceae were significantly higher in surface water samples collected when the aerators were off (Fig. 4). Notably, these bacteria are all obligate anaerobes (Yutin and Galperin 2013; Cruz-Morales et al. 2019). The relative abundance of the class Clostridia is also much higher when the aerators are turned off (Fig. 3, GW-A, PW-A, GW-B, PW-B) and LDA showed that this difference was statistically significant (Fig. 4). Note that GW-A and PW-A might have included CSO-related bacteria. While polyphyletic, the class Clostridia consists of obligate and aerotolerant anaerobes occupying a wide variety of ecological niches ranging from saprophytes to pathogens.

#### Campylobacteria

There was a high relative abundance of the class Campylobacteria in surface water samples taken in 2022 (GW-B, PW-B) compared to other samples (Fig. 3), and LDA showed that this difference was significant (Fig. 4). The majority of the Campylobacteria seen at these times belong to either the genus *Sulfurimonas* or the family Arcobacteraceae. *Sulfurimonas* is a metabolically versatile genus that is often found in a variety of marine environments (Han and Perner 2015). Additionally, Arcobacteraceae is found in association with sewage (Venâncio et al. 2022). A higher abundance of Campylobacteria is consistent with a possible decrease in DO after the aerators were deactivated, or Campylobacteria simply increased in abundance in 2022 compared to 2019.

### Taxa in dispersed foams versus aerosols

**Fig. 5:**
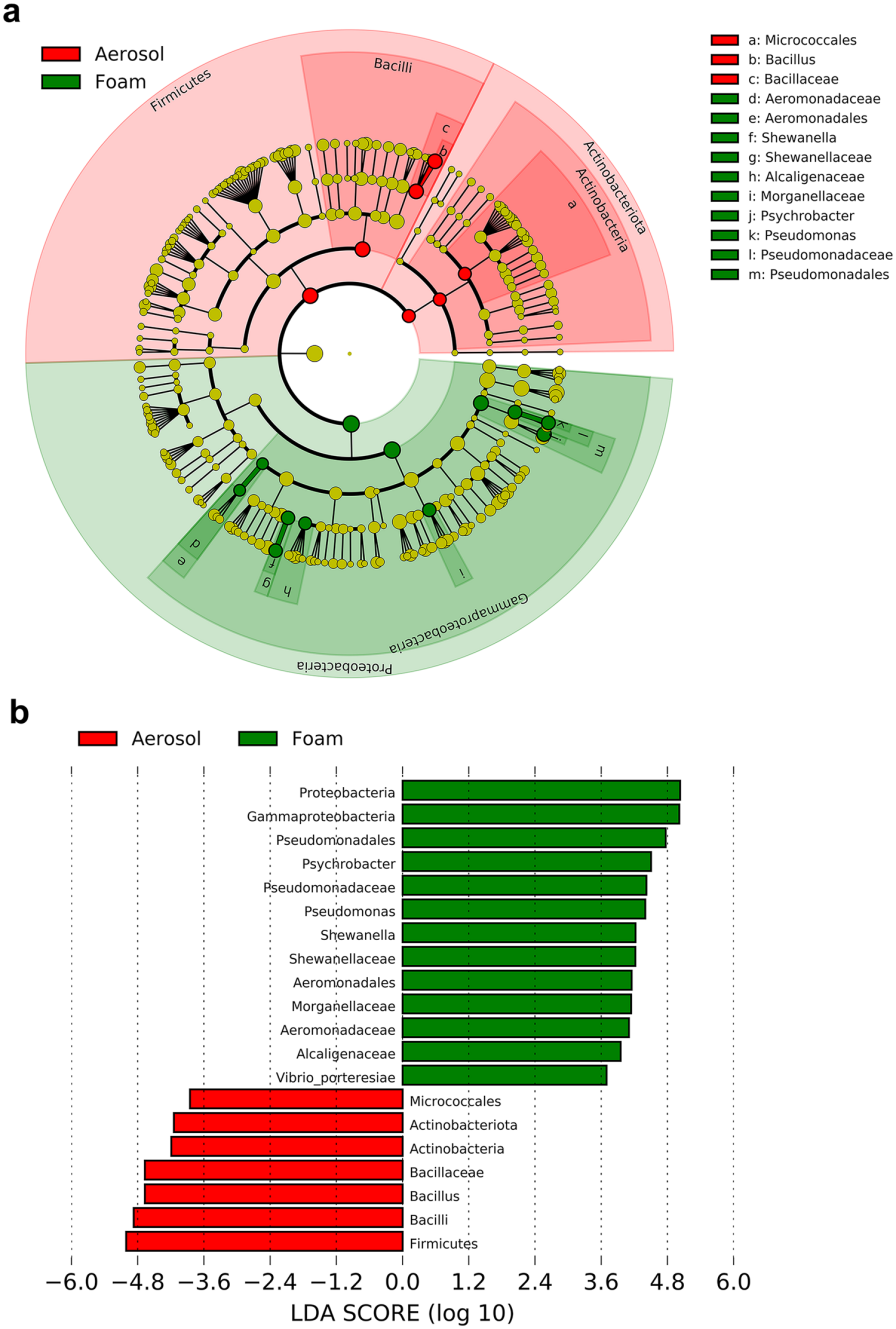
Linear discriminant analysis of effect size (LEfSe) of diversity of bacteria in plates grown from aerosol and foam. **a** Taxonomic representation of differential abundances of bacteria between aerosol and dispersed foam plates (LDA score > 2). **b** Histogram of LDA scores ranked by effect size

Collectively, Gram-positive bacteria (including Actinobacteria and Firmicutes) were more abundant in aerosols (see Fig. 5) whereas Gram-negative bacteria (including Gammaproteobacteria) were relatively more abundant in dispersed foam. The presence of biofilms, fimbria, and other cellular structures present in proteobacteria may contribute to the ready adherence of cells to the dispersed foam (Jin and Marshall 2020). Detailed analysis is included below.

#### Actinobacteria 

The detection of Actinobacteria in the aerosol and dispersed foam plates suggests that at least some Actinobacteria are being aerosolized by the aeration process. LDA showed that the order Micrococcales was significantly more abundant in the plates prepared from aerosols than from the dispersed foams (Fig. 5). The constituent genuses *Kocuria* and *Micrococcus* were detected in the plates. While both of these taxa are not typically pathogenic, they have been reported as opportunistic infections (Smith et al. 1999; Mattern and Ding 2014; Domont et al. 2014).

#### Firmicutes

The genus *Bacillus* was observed in the surface waters when the aerators were turned on but not with high relative abundance. However, it was detected in the plates cultured from aerosols at both sites. LDA shows that the higher abundance of the *Bacillus* in the aerosol plates was statistically significant in comparison to the dispersed foam plates. The type of aerators installed at the Creek are bottom-up in-stream aerators. As the forced air passes through and escapes from holes along the aerator pipes, it might disturb the sediment. Therefore, aeration may increase the possibility that benthic *Bacillus* would be dislodged from the bottom of the Creek, become resuspended, and aerosolized or become incorporated into foams. Several members of the genus *Bacillus* are potentially pathogenic and have been shown to spread via aerosol (Kuske et al. 2006; Merrill et al. 2006). There was a low relative abundance of Clostridia collected from aerosols as well as from dispersed foams. Of particular interest, although the genus *Clostridioides* was not found in the surface water samples, it was detected in plates grown from aerosols collected at Plank Rd. This genus includes pathogenic species such as *Clostridioides difficile* that can spread via the aerosolization of spores (Cooper et al. 2020). Although only a trace amount of the genus *Staphylococcus* was found in the surface water samples as well as in the dispersed foam plates, it was detected in the aerator plates. Previous studies have shown that some *Staphylococcus* can be dispersed by and survive in aerosols (Thompson et al. 2011).

#### Gammaproteobacteria

LDA showed that the higher relative abundance of several taxa within the class Gammaproteobacteria in dispersed foam plates when compared to aerosol plates was statistically significant. This included the constituent families Aeromonadaceae, Shewanellaceae, Alcaligenaceae, Morganellaceae, and Pseudomonadaceae. This is interesting because foam is formed when surfactant-containing water entraps air in bubbles, and foaming is a particular concern in aerated wastewater (Collivignarelli et al. 2020). Furthermore, foam production may be related to the ability of certain Gammaproteobacteria, including those of the constituent genuses *Aeromonas, Alcaligenes*, *Morganella, Pseudomonas*, and *Shewanella*, to produce and secrete biofilms (Wu et al. 2013; Hossain 2014; De et al. 2016; Talagrand-Reboul et al. 2017; Zorina et al. 2019). Foams may accumulate and pick up environmental microbes that are not part of the foam formation structure. Yet, the characteristics of foam movement require at least one or more organisms known to (a) produce biosurfactants to be able to form foam and (b) be highly hydrophobic to form stable foam (Guo et al. 2015). Further study may be required to elucidate the mechanism, if any, that microbes play in the formation of the dispersed foams.

While the genus *Escherichia* was detected at low relative abundance in surface waters and aerator foams, it was abundant in plates cultured from aerosols and dispersed foams. While we might expect *Escherichia* to be present in a water body, particularly one subject to CSO, the detection in aerosols and dispersed foams may be a concern as there are case reports that aerosolized *Escherichia* can cause illness (Graciaa et al. 2018; Ginn et al. 2021; Aluko et al. 2022).

The genus *Acinetobacter* was detected in all in situ samples (Table 3) and it was also detected in the cultured aerosol plates and dispersed foam plates. Members of this genus can become aerosolized and spread in hospital settings (Munoz-Price et al. 2013).

The genus *Stenotrophomonas* was detected on both aerosol and dispersed foam plates. While *Stenotrophomonas* is an aquatic taxon, some members of this group are opportunistic pathogens associated with pneumonia and aerosolized spread (Denton et al. 2003; Adegoke et al. 2017).

## Conclusions

Newtown Creek is a heavily polluted urban waterway in need of remediation. The Creek suffers from chronically low DO levels due to its constant inundation with CSOs as well as historic and ongoing industrial pollution. To address the anoxic environment, New York City DEP installed bottom-up in-stream aerators to increase DO into the waterway. This study suggests that the process of aeration may aerosolize pathogens and introduce new ones from sediments. When the aerators are on, the relative abundance of Actinobacteria and, specifically, the Microtrichales increases. Prior to the aerators being turned on 2019, Firmicutes and specifically Clostridia had a higher relative abundance, whereas Campylobacteria had a higher relative abundance in 2022 after the aerators had been off for 2 years (Table 3).

In this study, we identified potentially pathogenic bacteria in the surface water as well as the aerosols and the foam generated by the aerators. To our knowledge, this is the first report that identified the bacterial composition of the aerator foam and dispersed foam that forms at Newtown Creek when the aerators are turned on. Although several potential pathogens were noted, we cannot conclude that these taxa represent pathogens without further study. Newtown Creek is subject to continuing CSO pollution that inundates the Creek with sewage (Calderon et al. 2017). Thus, whether the process of aeration contributes to the dispersal of CSO-associated microorganisms and pathogens in the environment should be further studied.

## Data Availability

Metagenomic sequencing data is available at Zenodo repository 10.5281/zenodo.8277796.

## Footnotes

1 Tier 2 status is for the largest and most complex sediment sites containing over 7500 m^3^ of sediments (United States Environmental Protection Agency 2022).
